# The Harveian Society

**Published:** 1896-10-24

**Authors:** 


### The Harveian Society.

At the meeting held on October 15th, Mr. John Griffith read a paper, in which he collated and contrasted the various non-traumatic causes of sudden loss of sight. Underlying many of them were certain constitutional conditions, among which Bright's disease took the first place, although even in this disease sudden loss of sight was the exception, not the rule.

Sudden blindness might arise from uræmia, chiefly in acute inflammatory disorders of the kidney, and it was worth noting in such cases that, although the blindness might be absolute, the pupils continued to respond to light in 75 per cent. of the cases.

Hæmorrhage involving the macula lutea was by no means confined to Bright's disease, occurring as it did in connection with any constitutional predisposing cause. In some cases of sub-hyaloid hæmorrhage, in which the loss of sight was not complete, it gave rise to a sudden visual defect, in which objects appeared of a red colour. The author had seen several such cases in young male adults with perfect health.

Thrombosis of the central artery of the retina, or of the vein, and hæmorrhage into the optic-nerve-sheath in cases of cerebral hæmorrhage, were all causes of sudden blindness in renal disease, and tended to emphasise the fact that Bright's disease was the most common constitutional cause of sudden blindness, just as it was of sudden death.

The next cause of sudden loss of sight which was referred to was embolism of the central artery, which, when complete, is attended in most cases with sudden absolute and permanent blindness. It was pointed out, however, that there are exceptions to this rule of absolute loss of sight, abnormal branches sometimes being sufficient to maintain the circulation to a certain extent. Loss of sight from optic neuritis might seem to come on suddenly, although the disease might possibly have existed for some days or weeks before without declaring itself by interference with vision. Acute glaucoma was the malady which, above all others, caused sudden blindness. The ophthalmoscope was of no value in the diagnosis of this condition, for the media were too hazy and the details of the fundus too obscure. The disease might simulate a bilious attack, which, unfortunately, was a common error, leading to disastrous consequences. The points indicating glaucoma were: Congestion of the eyeball; dilated, oval, and fixed pupils; anæsthesia and steamy appearance of the cornea; hardness of the eyeball; great pain at the back of the eye, and also neuralgic pains along the course of the branches of the fifth nerve; prostration, reflex vomiting or retching, loss of appetite, and sleeplessness. The last local cause mentioned of sudden loss of sight was detachment of the retina, chiefly seen in cases of myopia, or of choroidal or retinal tumours.

Mr. Griffith illustrated his paper by an admirable series of lantern slides, many of which were lent by Mr. Adams Frost.

